# Incidence of intussusception before and after the introduction of rotavirus vaccine in Korea

**DOI:** 10.1371/journal.pone.0238185

**Published:** 2020-08-28

**Authors:** Hye-Kyung Cho, Se Hwan Hwang, Hye Na Nam, Kyungdo Han, Bongsung Kim, Insik Kong, Kwangsuk Park, Jaeyoung Lee

**Affiliations:** 1 Department of Pediatrics, Gil Medical Center, Gachon University College of Medicine, Incheon, Republic of Korea; 2 Department of Otolaryngology-Head and Neck Surgery, College of Medicine, The Catholic University of Korea, Seoul, Republic of Korea; 3 Department of Biostatistics, College of Medicine, The Catholic University of Korea, Seoul, Republic of Korea; 4 Department of Statistics and Actuarial Science, Soongsil University, Seoul, Republic of Korea; 5 Division of Vaccine-Preventable Disease Control and National Immunization Program, Korea Centers for Disease Control and Prevention, Cheongju, Republic of Korea; Public Health England, UNITED KINGDOM

## Abstract

**Background:**

Recent studies have reported that after the introduction of rotavirus vaccine the incidence of intussusception did not change among infants, or slightly increased at the age immediately after the first dose. The rotavirus vaccines were introduced in Korea for private market use in 2007–2008. We investigated the incidence of intussusception before (2002–2006) and after (2009–2015) the vaccine introduction in Korea.

**Methods:**

We conducted an interrupted time series study that used data from the Korean National Health Insurance database to identify infants (<12 months of age) who were diagnosed with intussusception and underwent non-invasive or invasive reduction from 2002 to 2015. According to the recommended ages for immunization, the annual intussusception incidence and the incidence rate ratios were calculated among three age groups, 6–14, 15–24, and 25–34 weeks.

**Results:**

The annual incidences in infants have decreased over time from 241.7 per 100,000 infants (pre-vaccine period) to 160.1–205.2 per 100,000 infants (post-vaccine period). The incidence rate ratio during the post-vaccine period ranged from 0.66 to 0.85. The incidences of intussusception in all three infant age groups have decreased in post-vaccine period compared to pre-vaccine period (incidence rate ratio range: 0.31–0.65, 0.47–0.75, and 0.68–0.94 in 6–14, 15–24, and 25–34 weeks, respectively).

**Conclusions:**

The incidence of intussusception in infants did not increase after the rotavirus vaccine introduction in Korea, but rather decreased over the past decades. Since the incidence of intussusception varies according to country or region, continuous monitoring the incidence of intussusception in infants is necessary in each county or region.

## Introduction

Intussusception is the most common cause of acute intestinal obstruction in infants and young children. Recent literature reviews have reported that the mean incidence of intussusception in infants was 74 per 100,000 infants, which significantly varied by country and region [1]. The incidence of intussusception in Asian countries, such as China, Japan, Vietnam, and Korea, has been much higher, ranged 100–300 per 100,000 infants, than that in the US and Latin America [1].

In 1999, the first vaccine against rotavirus (RotaShield; Wyeth Lederle, Philadelphia, PA, USA) was withdrawn due to its association with an increased risk of intussusception [2]. In the large pre-licensure trials of RotaTeq (Merck & Co. Inc., Whitehouse Station, NJ, USA) and Rotarix (GlaxoSmithKline Biologicals, Rixensart, Belgium), both vaccines did not increase the risk of intussusception and have been widely used [3, 4]. However, post-licensure population-based studies have shown conflicting results. Some cohort and case-control studies conducted in Australia, Brazil, Mexico, and the US reported the rate of intussusception after vaccination significantly increased, and the attributable risk was approximately 1–5 per 100,000 vaccinated infants [5–8]. Some ecological studies conducted in Australia, Canada, Taiwan, and the US have not shown an overall increase in the incidence of intussusception in infants [9–12]. In the studies conducted in Australia and the US, its incidence rate slightly increased at the age immediately after the first vaccination [8, 12].

Two globally licensed rotavirus vaccines (RVs) were introduced in Korea in June 2007 (RotaTeq) and March 2008 (Rotarix), which have been available only for private market use not for national immunization. At present, the exact vaccination coverage of the RV in Korea is not fully elucidated. However, the vaccine coverage based on the survey data from randomly selected infant populations was around 60% in the birth cohort born in 2010 [13].

We conducted an ecological study to examine the population-level trends of intussusception among infants in Korea from 2002 to 2015. This study aimed to investigate the trends in the incidence of intussusception before and after the introduction of the RV in children in Korea using the data of the National Health Insurance.

## Methods

### Study design

We conducted an interrupted time series study on infants (<12 months of age) with intussusception from January 1, 2002 to December 31, 2015 using the claims data of the Korean National Health Insurance Service (KNHIS). The KNHIS covers 100% of the population in Korea, including 97% on health insurance and 3% on medical aid. All clinics and hospitals in Korea submit information about their inpatients and outpatients, which include data on diagnoses (defined by the International Classification of Diseases, Tenth Revision [ICD-10]), treatment, demographic information, and medical costs for claims.

We obtained data on infants with intussusception between 2002 and 2015 from the KNHIS database. Then, the trends in the incidence of intussusception according to the recommended ages for rotavirus vaccination (6–14, 15–24, and 25–34 weeks) were investigated. Moreover, we compared the mean incidence rate of intussusception for pre-vaccine licensure years 2002–2006 with that for post-vaccine licensure years 2009–2015.

In this analysis, intussusception was defined as a patient who received inpatient or outpatient care with ICD-10 code K56.1 for intussusception and with the following procedure or operation codes for treatment of intussusception: G0300 (non-invasive reduction of intussusception in a child <8 years of age, successfully reduced), G0310 (non-invasive reduction of intussusception in a child <8 years of age, unsuccessful case), M6781 (non-invasive reduction of intussusception, successfully reduced), M6782 (non-invasive reduction of intussusception, unsuccessful case), Q2841 (manual operative reduction), Q2842 (resection with end-to-end anastomosis), Q2871 (operation of internal bowel hernia reduction), or Q2872 (operation of internal bowel hernia resection). We excluded patients with intussusception diagnosis code K56.1 without a procedure or operation code. If more than one episode of intussusception occurred in a patient, only the first case was included. The infant’s age in days, procedure and operation code, and date of diagnosis were obtained from the database.

This study was approved by the institutional review board of Gachon University Gil Medical Center (GCIRB2017-184).

### Statistical analysis

The incidence rates of intussusception per 100,000 infants were obtained by dividing the number of patients with intussusception by the number of population. The RV coverage rates were calculated by dividing the number of vaccinated infants registered on the national registry system by the number of population. The number of populations was based on infants registered in the KNHIS database as newborns born in each year. Births were assumed to be evenly distributed throughout the year when we calculated the rates by age groups. To compare the risk of intussusception and the hospitalization rates before and after the introduction of the RV, we calculated the incidence rate ratios (IRRs) and 95% confidence intervals (CIs) via Poisson regression analysis. All statistical analyses were performed using the SAS version 9.4 (SAS Institute Inc., Cary, NC, USA) based on a significance level of 0.05.

## Results

**Fig 1:**
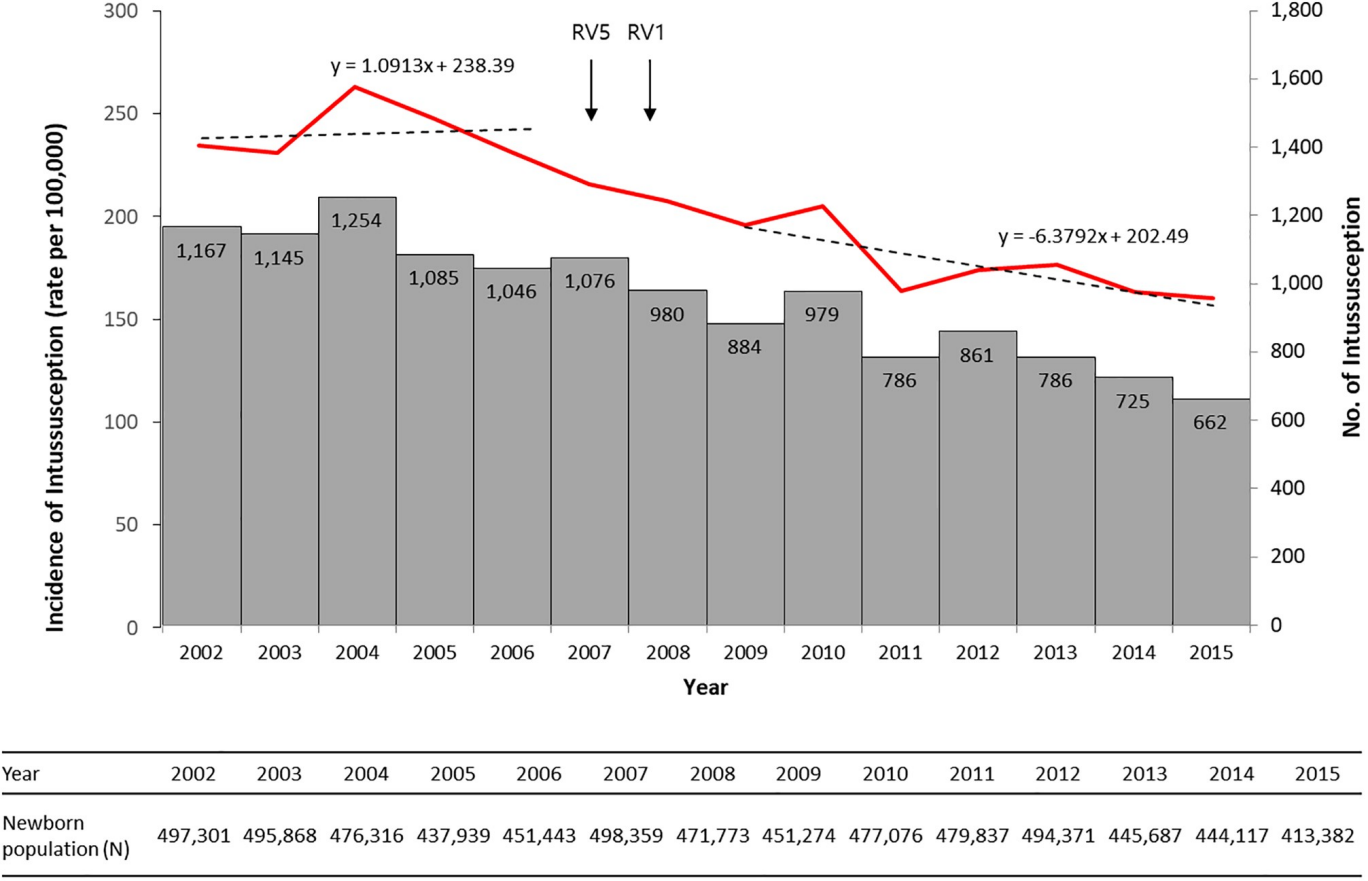
Trends in incidence of intussusception among infants <12 months of age in Korea, 2002–2015. The dotted lines indicate a linear fit for each of the pre- and post-vaccine periods (red line, incidence per 100,000 infants; bars, No. of intussusception; arrows, the time of vaccine introduction; RV5, pentavalent rotavirus vaccine, Rotateq; RV 1, monovalent rotavirus vaccine, Rotarix).

A total of 13,436 cases of intussusception occurred among infants in the Republic of Korea during the 14-year study period from 2002 to 2015. Annual numbers of intussusception and newborn population were shown in Fig 1. In 2002–2006, before the introduction of the RV in Korea, the annual incidence of intussusception in infants was relatively constant annually, ranging from 230.9 per 100,000 infants in 2003 to 263.3 per 100,000 infants in 2004, and the mean incidence was 241.7 per 100,000 infants (Fig 1, Table 1). During the post-vaccine era, the incidence of intussusception significantly reduced over time (Fig 1) and the mean incidence was 177.3 per 100,000 infants (IRR: 0.73; 95% CI: 0.71–0.76), ranging from 205.2 per 100,000 infants (IRR: 0.85; 95% CI: 0.79–0.91) in 2010 to 160.1 per 100,000 infants in 2015 (IRR: 0.66; 95% CI: 0.61–0.72) compared to the mean incidence during the pre-vaccine period (Table 1).

**Table 1:** Incidence of intussusception per 100,000 infants by age group in the pre- and post-rotavirus vaccine years (2002–2006 and 2009–2015, respectively).

Age group	2002–2006	2009–2015		2009		2010		2011		2012		2013		2014		2015	
	Mean rate (min, max)	Mean rate (min, max)	IRR (95% CI)	Rate	IRR (95% CI)	Rate	IRR (95% CI)	Rate	IRR (95% CI)	Rate	IRR (95% CI)	Rate	IRR (95% CI)	Rate	IRR (95% CI)	Rate	IRR (95% CI)
<12 mo	241.7 (230.9, 263.3)	177.3 (160.1, 205.2)	0.73 (0.71, 0.76)	195.9	0.81 (0.76, 0.87)	205.2	0.85 (0.79, 0.91)	163.8	0.68 (0.63, 0.73)	174.2	0.72 (0.67, 0.77)	176.4	0.73 (0.68, 0.79)	163.2	0.68 (0.63, 0.73)	160.1	0.66 (0.61, 0.72)
6–14 wk	90.8 (83.4, 98.1)	43.2 (27.8, 40.6)	0.48 (0.40, 0.56)	59.1	0.65 (0.48, 0.88)	48.6	0.53 (0.39, 0.74)	27.8	0.31 (0.20, 0.47)	48.0	0.53 (0.38, 0.73)	32.5	0.36 (0.24, 0.54)	45.7	0.50 (0.36, 0.71)	40.6	0.45 (0.31, 0.65)
15–24 wk	256.8 (222.9, 310.9)	157.2 (129.9, 193.5)	0.61 (0.56, 0.66)	180.3	0.70 (0.59, 0.83)	193.5	0.75 (0.64, 0.88)	121.7	0.47 (0.39, 0.57)	145.6	0.56 (0.47, 0.67)	174.3	0.68 (0.57, 0.80)	153.8	0.60 (0.50, 0.72)	129.9	0.50 (0.41, 0.62)
25–34 wk	315.7 (292.2, 340.5)	243.1 (213.2, 296.2)	0.77 (0.72, 0.83)	239.2	0.76 (0.65, 0.88)	296.2	0.94 (0.82, 1.07)	220.6	0.70 (0.60, 0.81)	232.0	0.73 (0.64, 0.85)	272.6	0.86 (0.75, 0.99)	224.2	0.71 (0.61, 0.83)	213.2	0.68 (0.58, 0.79)

IRR, incidence rate ratio; CI, confidence interval.

**Fig 2:**
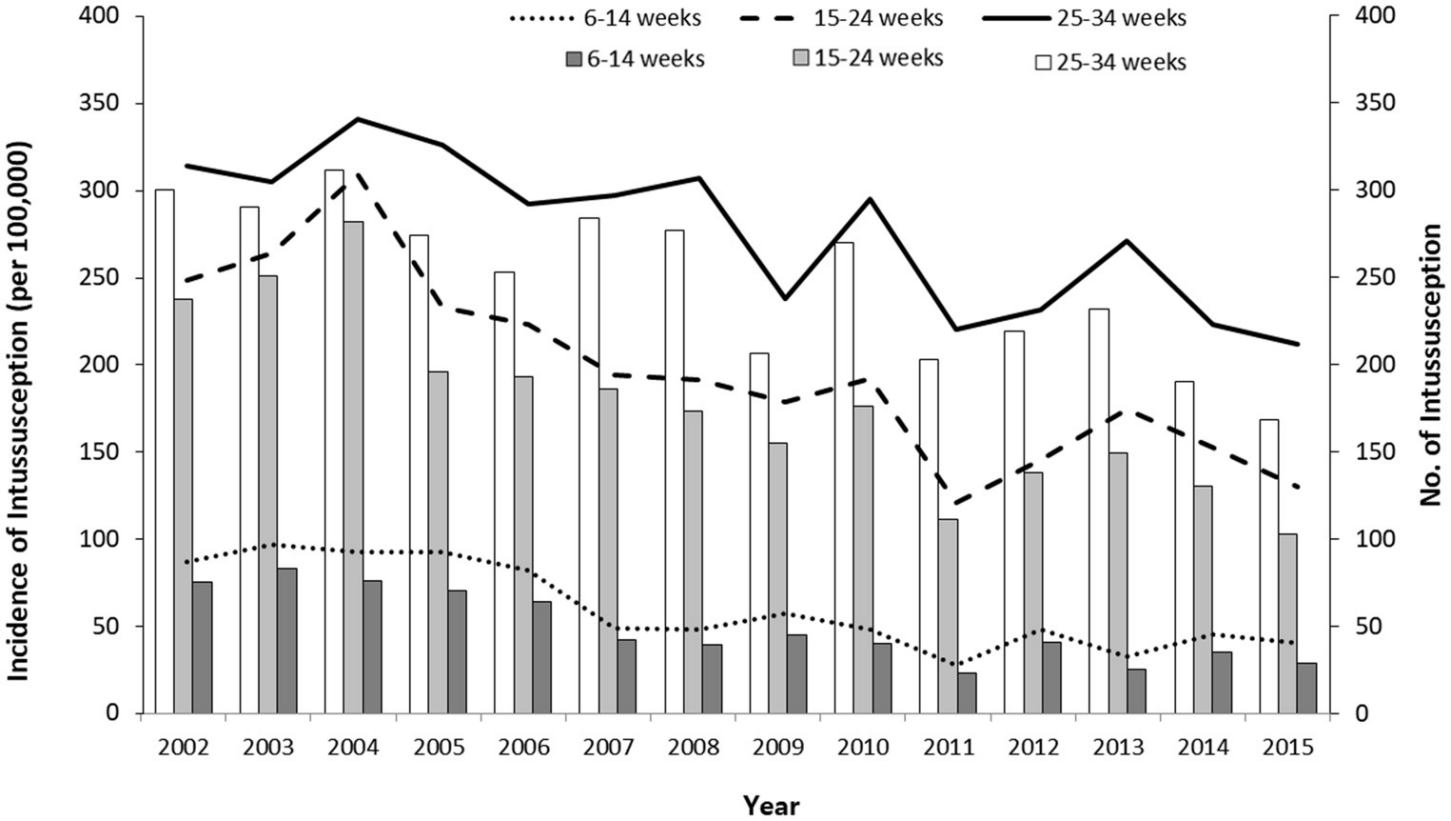
Trends in the incidence of intussusception between 2002 and 2015 by infant age groups (6–14, 15–24, and 25–34 weeks) based on the recommended ages for the rotavirus vaccine in Korea (lines, incidence per 100,000 infants; bars, No. of intussusception).

The incidence of intussusception in all three infant age groups (6–14, 15–24, and 25–34 weeks) decreased in post-vaccine period compared to pre-vaccine period. It was lowest in infants aged 6–14 weeks during the entire study period, followed by those aged 15–24 and 25–34 weeks (Fig 2 and Table 1). During the pre-vaccine period, the mean incidence of intussusception in infants aged 6–14 weeks was 90.8 per 100,000 (range: 83.4–98.1 per 100,000) infants. In infants from this age group, the decrease in the incidence rate was most evident during the post-vaccine period. The rate reduced by 35% in 2009 (59.1 per 100,000 infants; IRR: 0.65; 95% CI: 0.48–0.88), 69% in 2011 (27.8 per 100,000 infants; IRR: 0.31; 95% CI: 0.20–0.47), 50% in 2014 (45.7 per 100,000 infants; IRR: 0.50; 95% CI: 0.36–0.71), and 55% in 2015 (40.6 per 100,000 infants; IRR: 0.45; 95% CI: 0.31–0.65). The changes in the incidence rate of intussusception in infants aged 15–24 and 25–34 weeks were similar. During the pre-vaccine era, the mean rate of intussusception in infants aged 15–24 weeks was 256.8 per 100,000 (range: 222.9–310.9 per 100,000) infants. During the post-vaccine period, the annual rate of intussusception in this age group decreased by 25–53% (range: 121.7–193.5 per 100,000 infants). In infants aged 25–34 weeks, the mean rate of intussusception during the pre-vaccine period was 315.7 per 100,000 infants, and the annual incidence rate of intussusception during the post-vaccine period decreased by 6–32% (range: 213.2–296.2 per 100,000 infants), except in 2010 (296.2 per 100,000 infants; IRR: 0.94; 95% CI: 0.82–1.07), when the incidence rates of intussusception did not significantly decrease.

The mean rate of hospitalized infants with intussusception in the post-vaccine period was 73.3%, which lower than that in the pre-vaccine period (77.1%; IRR: 0.95; 95% CI: 0.91–0.99). Compared with the pre-vaccine period, the proportion of patients treated with surgery reduced after the introduction of RV and further decreased over time. The rate of surgical treatment during the pre-vaccine period ranged from 10.0% to 14.2%. However, it only ranged from 7.2% to 10.0% in 2009–2012 and from 6.2% to 6.6% in 2013–2015 after the introduction of the vaccine.

## Discussion

The risk of intussusception associated with rotavirus vaccination is an important safety issue in determining vaccine policy. Several countries have carefully monitored the occurrence of intussusception after vaccination, and others that have not yet introduced the vaccine are collecting baseline data on the incidence of intussusception before vaccination [14, 15]. The pathogenesis of intussusception associated with RV is not fully elucidated. Moreover, it is also unclear which cases should be considered as vaccine-related adverse events based on the time elapsed from vaccination to intussusception or their distinct characteristics. Several studies investigating the risk of intussusception associated with RV have focused on its incidence in young infants [10–12, 16, 17]. In studies conducted in the US, Canada, and Taiwan, no changes were observed in the incidence of intussusception in infants before and after the implementation of the vaccine [10–12]. In the present study, the incidence of intussusception after the introduction of the RV did not increase, but rather decreased in Korea. A subset analysis of infants enrolled in the clinical trial of a RV that had followed up until 12 months of age in Latin America reported a reduction in the risk of intussusception among vaccine recipients compared with placebo recipients (relative risk: 0.28; 95% CI: 0.10–0.81) [18]. However, there have been few studies reporting reduced risk at the population level. A Taiwanese data showed that hospitalization rates of intussusception in infants were lower during post-vaccine period than during pre-vaccine period [10], which is consistent with the data of the present study. According to a literature review, any potential risk of intussusception in populations with higher baseline risk may lead to a higher number of excess cases that would be attributable to the vaccine [1]. However, data from Korea and Taiwan, where are considered to have higher baseline risk, showed that the incidence of intussusception in the population of infants has decreased after vaccine introduction [10].

Although data on the incidence of intussusception in Korea has been extremely limited, the baseline incidence of intussusception among infants before the introduction of the vaccine was significantly higher (328 per 100,000 infants) than other countries [1, 19]. Data from a literature review have also shown that the incidence of intussusception in Asian countries, such as Japan and Vietnam (185 and 302 per 100,000 infants, respectively), was higher than that in American and European countries (20–72 per 100,000 infants). However, the reason for this was unknown [1]. These differences were attributed to genetic predisposition, environmental differences, including circulating pathogens, dietary pattern of infants, breastfeeding, and accessibility to medical care [20, 21].

**Fig 3:**
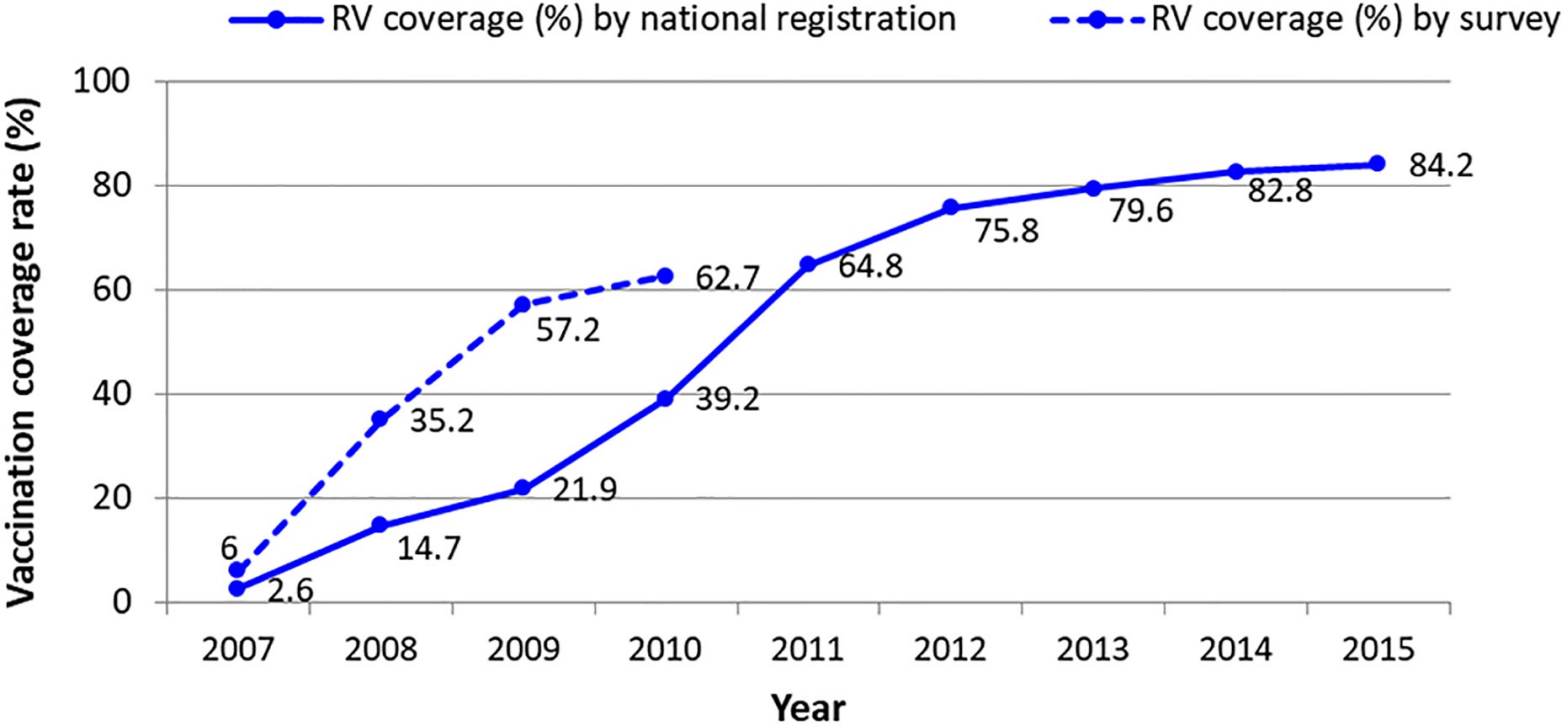
Rotavirus vaccine coverage rate between 2007 and 2015. Solid line indicates the rotavirus vaccination coverage (≥ 1 dose) from national immunization registry of Korea (Korea Centers for Disease Prevention and Control, unpublished data) and dotted line indicates the coverage from nationwide survey of immunization rate (Korea Centers for Disease Prevention and Control, National Immunization Survey 2011–2014, unpublished data).

The decreasing incidence of intussusception in infants in Korea after the introduction of RV might be associated with some factors. First, it could be assumed that a decrease of wild-type rotavirus infection would have had an impact on the incidence of intussusception. The RV coverage in Korea has steadily increased since the introduction of RV up to 84.2% in 2015 (Korea Centers for Disease Prevention and Control, National Immunization Survey 2011–2014 and National Immunization Registry, unpublished data, Fig 3). In Fig 3, the coverage rates from the registry were lower than those from the survey at the beginning of RV introduction, and the gap between those two rates narrowed over time. Since the establishment of the registry in 2009, it has not been mandatory for healthcare provider to register the RV uptake of infants because RV is a non-routine vaccine in Korea. As more kinds of vaccines, such as *Haemophlius influenzae* type b vaccine in 2013, pneumococcal conjugate vaccine in 2014, and hepatitis A vaccine in 2015, have been added to routine vaccines, more non-routine vaccination records, including RVs, may have been registered in the registry. Nevertheless, RV coverage may be underestimated since all records may not have been registered. Although data on the timeliness of rotavirus vaccination in Korea are unknown, it was estimated to be about 90% from that of the diphtheria-tetanus-acellular pertussis vaccine, a routine vaccine inoculated at a schedule similar to RV [22].

Meanwhile, the incidence of rotavirus infections has markedly decreased among young infants [23–25]. Although it is still controversial whether rotavirus, both wild-type and vaccine strain, is associated with the development of intussusception [26], it could be an attributable factor to this decreasing trend of intussusception. Some studies that have denied association between intussusception and rotavirus infection have implemented detecting rotavirus in fecal samples of patients with intussusception [26] or comparing seasonality between these two diseases [27, 28]. However, the pathogenesis of intussusception is not clearly elucidated. In a study that measured ileum wall thickness and mesenteric lymph nodes using ultrasound in infants with rotavirus infection and healthy controls, increased ileum wall thickness and lymph node size were identified in infants with rotavirus infection [29]. More researches are still needed to determine the association between intussusception and rotavirus infection.

Second, it is possible that changes in people’s health behaviors, dietary patterns of infants or other socio-environmental factors have affected epidemiological changes of other infections, reducing the incidence of intussusception in infants. Although the causes of intussusception are unknown in most cases and the pathogenesis of intussusception is unclear, several studies suggested some infectious pathogens, such as respiratory adenovirus and some enteric bacteria, were associated with its occurrence [30–34]. In Fig 1, the incidence of intussusception also seems to have started to decline before the introduction of RV, suggesting that it may be more related to changes in factors other than vaccines. If the incidence data prior to 2002 was available, the epidemiology of the intussusception in the pre-vaccine era could have been more clearly understood, but unfortunately the KNHIS database was not able to provide them.

In this study, the incidence of intussusception in all infant age groups has decreased after the introduction of the vaccine. The incidence decreased the most in infants aged 6–14 weeks (at the age of first dose), followed by those aged 15–24 weeks (second dose) and 25–34 weeks (third dose). In studies conducted in the US and Australia, the incidence of intussusception slightly increased in infants at the age of the first dose and the second dose, respectively [8, 12]. In a study conducted in Canada, those did not change in infants of all age groups [11]. In Taiwan, it did not change in infants at the age of the first and second dose, but decreased in infants at the age of the third dose during the post-vaccine era [10]. Since there are differences in the epidemiologic change of intussusception after the introduction of vaccines by country or region, it is necessary to monitor the incidence of intussusception in infants where RVs are being used or planned to be introduced.

This study showed the ecologic data suggesting that the incidence of intussusception has been decreasing after the introduction of the RV in Korea. This study can be a representative of the whole infant population of the nation regardless of income level or region and can include both outpatient and inpatient data from the KNHIS database.

This study has some limitations. First, we included the cases with both the diagnostic code for intussusception and the treatment code for non-surgical or surgical reduction. Although the inclusion of only the treated intussusception cases was not validated, it was possible to verify their diagnosis because almost cases of intussusception need reduction non-surgically or surgically right after diagnosis, keeping its incidence from being overestimated. Also, since the diagnosis and treatment codes on the KNHIS system have not changed throughout the 15-year study period, it is reasonable to identify trends of the incidence rate during the study period. Even with tighter criteria, the incidence of intussusception in this study was higher than that of other countries [10–12]. Second, we could not investigate the serious outcomes, such as complications, sequelae or deaths, during the period of illness. It was impossible to extract such information from the database. Third, the national immunization registry system has not been directly linked with the KNHIS database and the vaccination records from individual infants with intussusception were unavailable. Therefore, we could not know if they had been vaccinated and it was not possible to analyze the difference between the incidence of intussusception among the vaccinated and non-vaccinated infants.

This study presented data on the incidence of intussusception before and after the introduction of RV in Korea, which can be used as the baseline data before the introduction of the RV into the national immunization program and as a safety data of RV. The incidence of intussusception in infants has decreased in Korea over the past decades regardless of the age category considering the vaccination time. This reduction might be due to the effect of vaccines in countries with high vaccine coverage and high baseline incidence of intussusception, but may have been affected by changes in other factors. Since the incidence of intussusception varies according to country or region, continuous monitoring the incidence of intussusception in infants is necessary in each country or region.

## Supporting information

S1 Table Annual number of population and intussusception cases among infants, 2002–2015. (XLSX)

S2 Table Annual incidence rate of intussusception in post-vaccine period (2009–2015) compared to that in prevaccine period (2002–2006). (XLSX)

S3 Table Number of procedure or surgery for treatment of intussusception. (XLSX)
